# Implantable bone conduction devices enhance functional ear reconstruction in patients with pediatric microtia

**DOI:** 10.3389/fped.2025.1614778

**Published:** 2025-07-17

**Authors:** Ying Chen, Chen-long Li, Liu-Jie Ren, Xu Wu, Na Gao, Ai-juan He, Yao-yao Fu, Ya-ying Zhu, Tian-yu Zhang

**Affiliations:** ^1^ Department of Facial Plastic and Reconstructive Surgery, Eye & ENT Hospital, Fudan University, Shanghai, China; ^2^ ENT Institute, Eye & ENT Hospital, Fudan University, Shanghai, China; ^3^ Department of ENT, Eye & ENT Hospital, Fudan University, Shanghai, China; ^4^ NHC Key Laboratory of Hearing Medicine, Fudan University, Shanghai, China

**Keywords:** microtia, hearing loss, auricle reconstruction, bone conduction implant, surgery

## Abstract

**Purpose:**

This study evaluated the audiometric outcomes and complications of simultaneous bone conduction device (BCD) implantation and auricular reconstruction, and the comparative effectiveness of three BCDs.

**Methods:**

In total, 41 patients with bilateral microtia (ranging from 8 to 16 years old) who underwent combined surgery from January 2018 to January 2024 were retrospectively analyzed. Audiometric parameters (free-field thresholds and speech recognition scores) and complications were compared across the groups of patients who received the Baha Attract (*n* = 13), Sophono (*n* = 18), and Bonebridge (*n* = 10) devices.

**Results:**

Significant improvements occurred in aided vs. unaided conditions, with mean free-field threshold values of 57.6 ± 7.42 vs. 22.19 ± 6.40 dB (*p* < 0.001) and speech recognition threshold values of 69.42 ± 4.21 vs. 39.16 ± 6.78 dB (*p* < 0.001). No significant inter-group differences emerged in hearing gain (*p* > 0.05). Device-related complications included transient skin reactions, hematoma, and pressure erythema, and all were resolved conservatively. The patients’ hearing threshold in a free field and speech recognition in quiet tests significantly improved after being implanted with the Baha Attract, Sophono, or Bonebridge hearing aid. There was no significant difference in hearing gain across frequencies between the subgroups. No patient in any of the subgroups reported major adverse events that affected the safety of the reconstructed auricle or the implant after the combined surgery.

**Conclusion:**

The three implants demonstrated satisfactory hearing performance in children with bilateral microtia. Combined BCD implantation and auricle reconstruction surgery was shown to be safe and effective.

## 1 Introduction

Microtia is a birth defect referring to the underdevelopment of the auricle, with prevalence rates ranging between 0.83 and 4.34 per 10,000 births (1). Usually, microtia is associated with congenital conductive hearing loss resulting from accompanying external ear canal stenosis/ atresia and a malformed middle ear. Children with bilateral microtia experience both esthetic concerns and hearing impairment and require plastic surgeries to correct the auricle anomalies and rehabilitate their hearing (2). Without early treatment of bilateral hearing loss, affected children may suffer from speech impairments and delayed language development.

Many clinical trials have shown that bone conduction devices (BCDs) are an effective way of restoring hearing in individuals with conductive or mixed hearing loss (3, 4). BCDs convert sound signals into vibration and stimulate the cochlea via the bone conduction pathway. BCDs provide ideal hearing outcomes for patients with bilateral microtia as they bypass the middle ear to improve hearing (5). The International Microtia and Atresia Workgroup strongly recommended bone conduction technology for children with bilateral aural atresia (6). Patients can wear BCDs using softbands or headbands, or they can be implanted at the appropriate age.

To provide optimal treatment that results in a “functional ear” for patients with bilateral microtia, many factors should be considered, including concordant multidisciplinary treatment, fewer surgery stages, and choosing a safe and effective BCD implant. For this reason, surgeons combine auricular reconstruction procedures with bone conduction device implantation to reduce the number of operation stages and the associated cost (7). Given that the mastoid in patients with microtia may be poorly developed, the extremely small mastoid space makes it difficult to choose a BCD implant. Furthermore, guaranteeing the safety of both the implant and the reconstructed ear remains a great challenge for surgeons. Although a few studies have reported the technique for the combined surgery and its clinical outcomes (8, 9), studies on how different BCD implants affect hearing outcomes and reconstructed ear safety have yet to be conducted.

In this study, we review and demonstrate the combined surgery technique, and also compare the acoustic outcomes and complications after simultaneous BCD implantation and auricle reconstruction surgery in children with congenital microtia with three different BCDs. The results of this study may provide some information to help clinicians during decision-making and conducting surgical procedures in children with microtia.

## 2 Materials and methods

### 2.1 Study setting and patient enrollment

This study was a single-center retrospective study. Pediatric patients who underwent unilateral BCD implantation alongside auricle reconstruction in our center from 2018 to 2024 were enrolled. The implantation criteria were as follows: (1) bilateral hearing loss; (2) the ear on the implant side had atresia with conductive hearing loss or a mainly conductive component, confirmed by pure tone audiometry preoperatively; (3) without indication for canal reconstruction.

### 2.2 Hearing threshold in a free-field test

Audiometry for all patients was performed in a standard soundproof booth before surgeries (unaided situation) and after device fitting (aided situation). Unaided and aided hearing thresholds were measured using a warble tone emitted by a speaker 1 m in front of the subjects. The untested ear was masked by an unilateral ear muff. Unaided and aided hearing threshold averages at the 0.5, 1, 2, and 3 kHz frequencies in a free field were evaluated.

### 2.3 Speech recognition

The speech recognition threshold (SRT) in quiet was measured using the Mandarin speech test, which was presented to the front side of the participants with a random list of 15 sentences. Each sentence consisted of seven Chinese characters (seven syllables). The software automatically logged the SRT threshold as half of the characters being correctly repeated in each list.

### 2.4 Surgery and fitting

**Figure 1:**
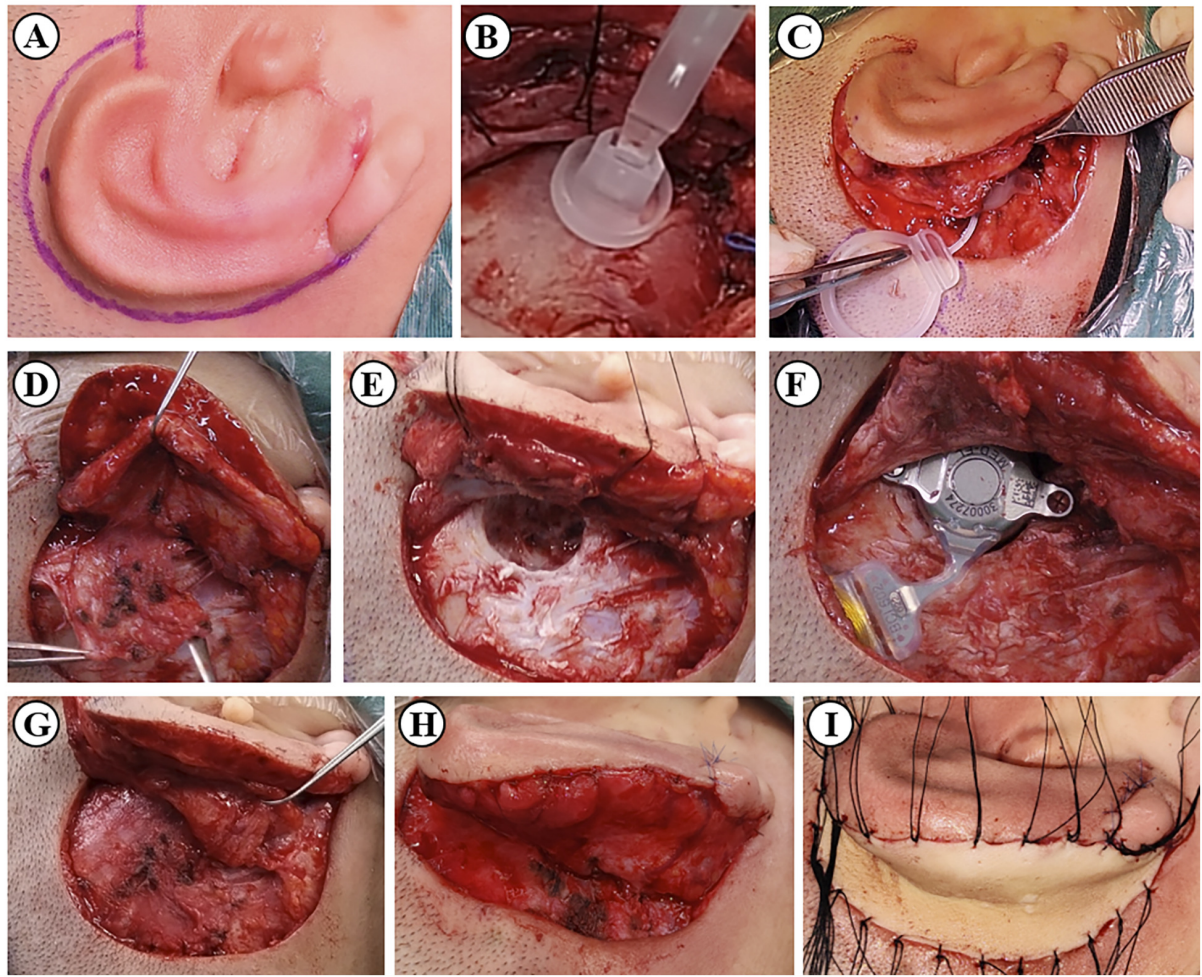
BCD implantation procedure combined with auricle framework elevation (Bonebridge implantation as an example). **(A)** The plan for the incision. **(B)** Location of the bone conduction-floating mass transducer using a model. **(C)** Location of the signal processor. **(D)** Dissection of the retroauricular fascial ﬂap and the periosteal flap. **(E)** Drilling the bone bed for the implant. **(F)** Fixation of the implant. **(G)** Closure of the periosteal flap. **(H)** Closure of the retroauricular fascial ﬂap. **(I)** Transplantation of the skin graft from the abdomen or scalp.

All patients had the *in vivo* part of the bone conduction device implanted unilaterally and underwent two-stage microtia surgery (auricle framework elevation) under general anesthesia. The implants were selected according to the patient's disease condition, preoperative hearing, mastoid volume according to a CT examination, and the patient's preference. The implantation was performed on the side with better bone conduction hearing or according to the patient's preference when bone conduction hearing was equal bilaterally. Incisions were first made 5 mm outside the posterior margin of the reconstructed auricle (Figure 1A). The superﬁcial layer of the fascia covering the dorsal part of the reconstructed auricle was dissected to the posterior edge of the cavum conchae. Then, 20 mm behind the incision, the retroauricular fascial ﬂaps were dissected and lifted to match the cranio-auricular sulcus. After lifting the retroauricular fascia, the periosteum at the implanted site was sufficiently exposed.

Considering that the retroauricular fascia flaps were moved anteriorly to cover the prosthesis for the ear evaluation, the surfaces of the implants were covered with a periosteal flap with the tip located in the posterior sulcus of the ear to guarantee the safety of the implantation (Figure 1G).

Then, the three aforementioned hearing aid implants were implanted using the standard surgical procedure, ensuring that there was no touching between the implant and any prominent surrounding bone and that the thickness of the flap above the implant was sufficient (Figure 1F).

Afterward, carved wedges of porous polyethylene or C-shaped autologous costal cartilage were used as the projection material in the reconstructed auricle. The retroauricular fascia flap was rotated to wrap the exposed part of the completed auricular framework (Figure 1H). Skin grafts were used to cover the posterior surface of the skin defect (Figure 1I).

Four weeks after surgery, when the wound had healed, one of the three BCD sound processors of BCDs were coupled to the implant and fitted.

### 2.5 Complications

Intra- and post-surgery complications were monitored by senior otologists. Each patient's guardian was educated on maintaining the implant and advised to report any adverse events after fitting.

### 2.6 Statistical analysis

The pure tone average (PTA) is the mean of the hearing threshold for the 0.5, 1, 2 and 3 kHz frequencies in a free field Hearing gain was the difference between unaided and aided PTA in the free-field test. Differences in acoustic outcome between unaided and aided situations were measured using a paired sample t-test or non-parametric permutation test using SPSS (SPSS Inc., v20.0, Chicago, IL, USA). The power was set to 95%. *P*-values <0.05 were considered statistically significant.

## 3 Results

### 3.1 Demography and surgery

**Figure 2:**
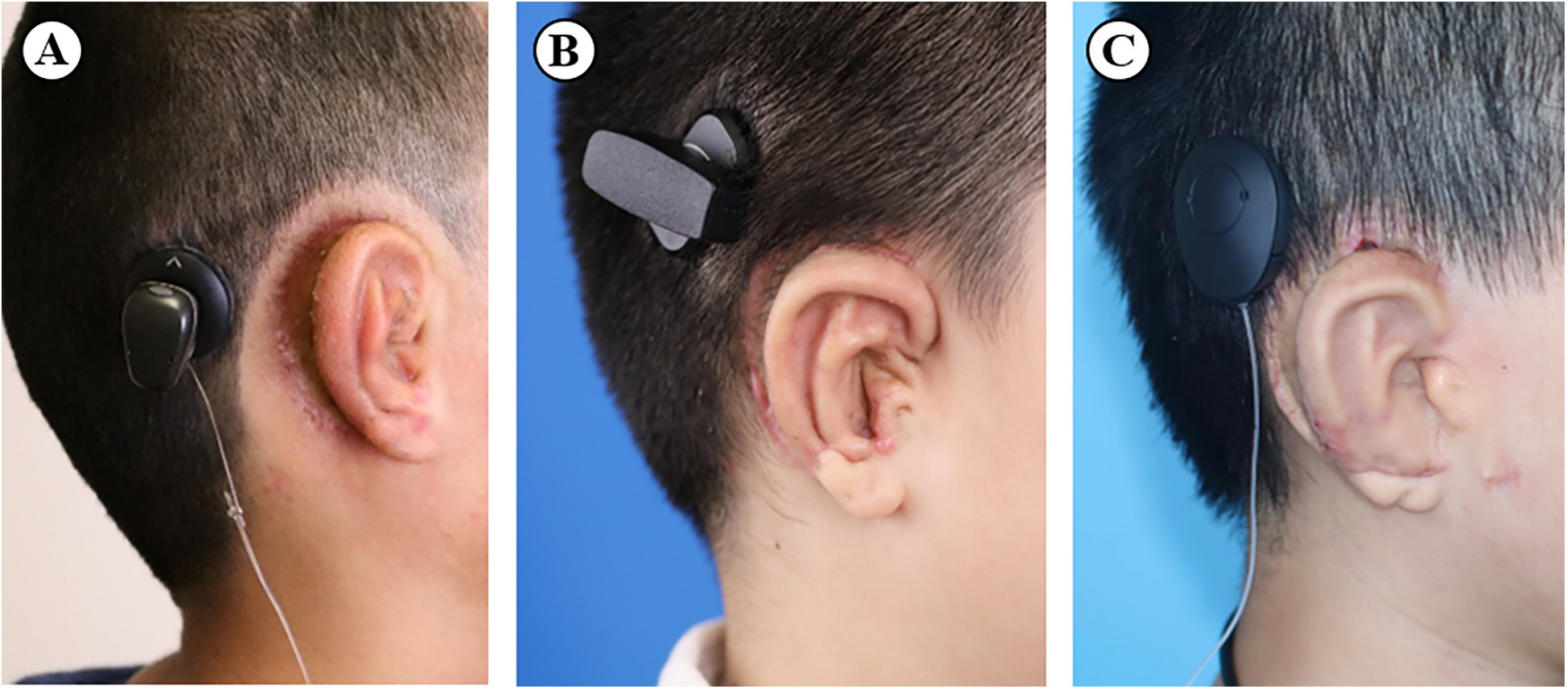
The final appearance of the **(A)** Baha Attract, **(B)** Sophono, and **(C)** Bonebridge after the combined surgery.

In total, 41 children were included in the present study, including 30 boys and 11 girls. The mean age was 10.76 ± 2.14 years, ranging from 8 to 16 years. The overall preoperative bone conduction hearing threshold average was 21.13 ± 8.46 dB in hearing level (HL), and was not significantly different between any two groups. The patients were followed up for 4 to 37 months. In this cohort, 13 cases were implanted with Baha Attract, 18 cases with Sophono, and the remaining 10 cases with Bonebridge. All cases received BCD implantation after auricle framework elevation at the same stage of surgery, with 26 cases receiving autologous costal cartilage as the projection material and the remaining 15 cases using an artificial material. The patient had esthetic improvement after the surgery, and the positions of the implants were proper (Figure 2).

### 3.2 Hearing threshold

**Figure 3:**
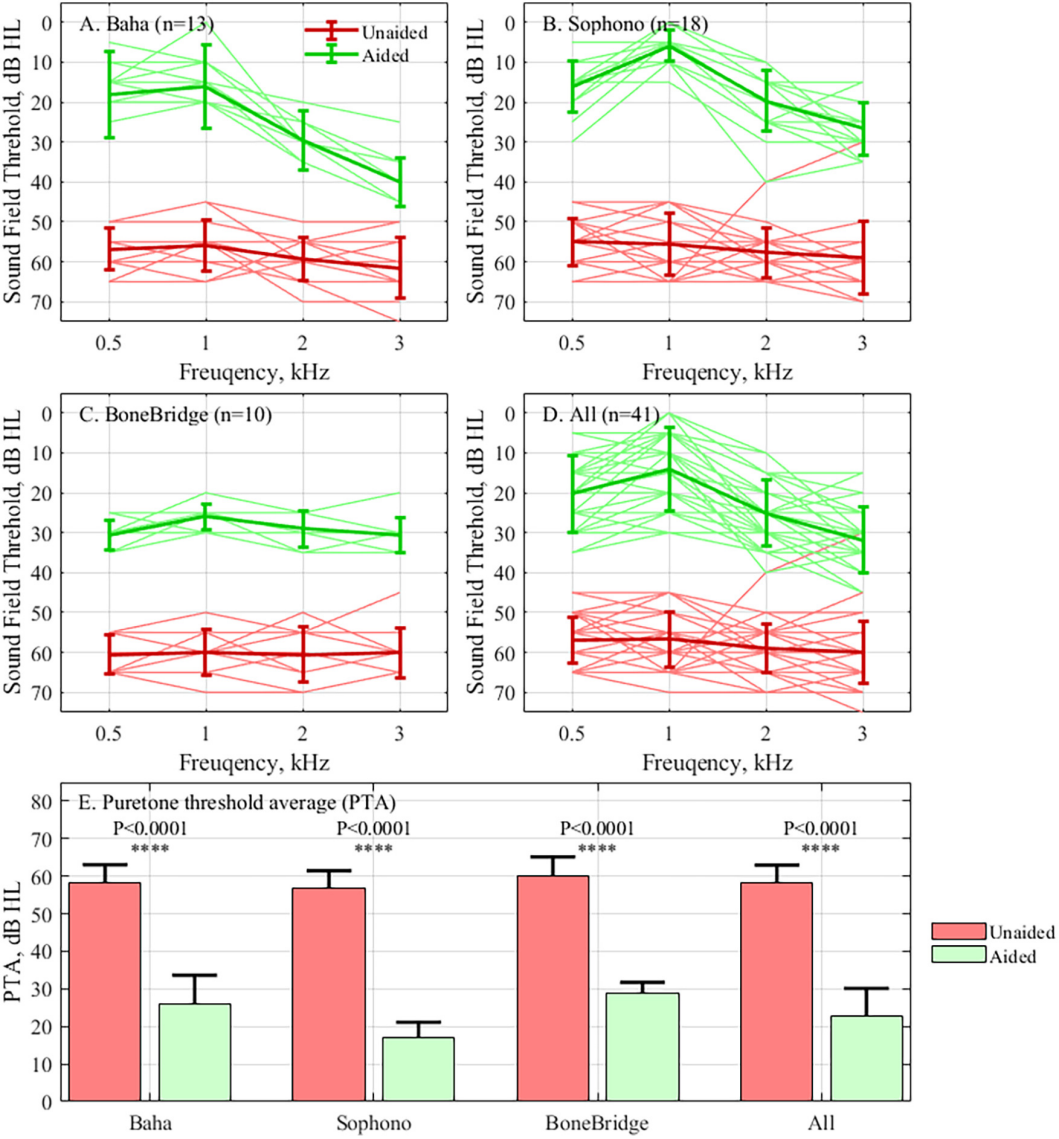
The hearing threshold test results. The results are presented as a frequency for the **(A)** Baha Attract subgroup, **(B)** Sophono subgroup, **(C)** Bonebridge subgroup, **(D)** All individuals, and **(E)** The means of unaided and aided PTA of different subgroups and all patients. The results from the unaided condition are shown in red and the aided condition in green.

The mean unaided and aided hearing threshold average of all participants was 57.60 ± 7.42, and 22.19 ± 6.40 dB SPL, respectively, demonstrating that implantable BCDs can effectively improve the hearing of bilateral microtia patients (*p* < 0.001). Unaided PTAs of the Baha Attract, Sophono, and Bonebridge groups were 58.02 ± 4.75, 55.42 ± 8.95, and 60.25 ± 4.92 dB SPL, respectively, which were reduced to 25.28 ± 3.23, 17.08 ± 4.07, and 29.00 ± 2.75 dB in sound pressure level (SPL) in the aided situation. Interestingly, each group's aided and unaided PTA averages were not significantly different (*p* > 0.05). The hearing thresholds and hearing gain at each frequency of all the participants and the three subgroups are shown in Figure 3. Hearing gain at each frequency showed no significant differences among the subgroups.

### 3.3 Speech recognition

**Figure 4:**
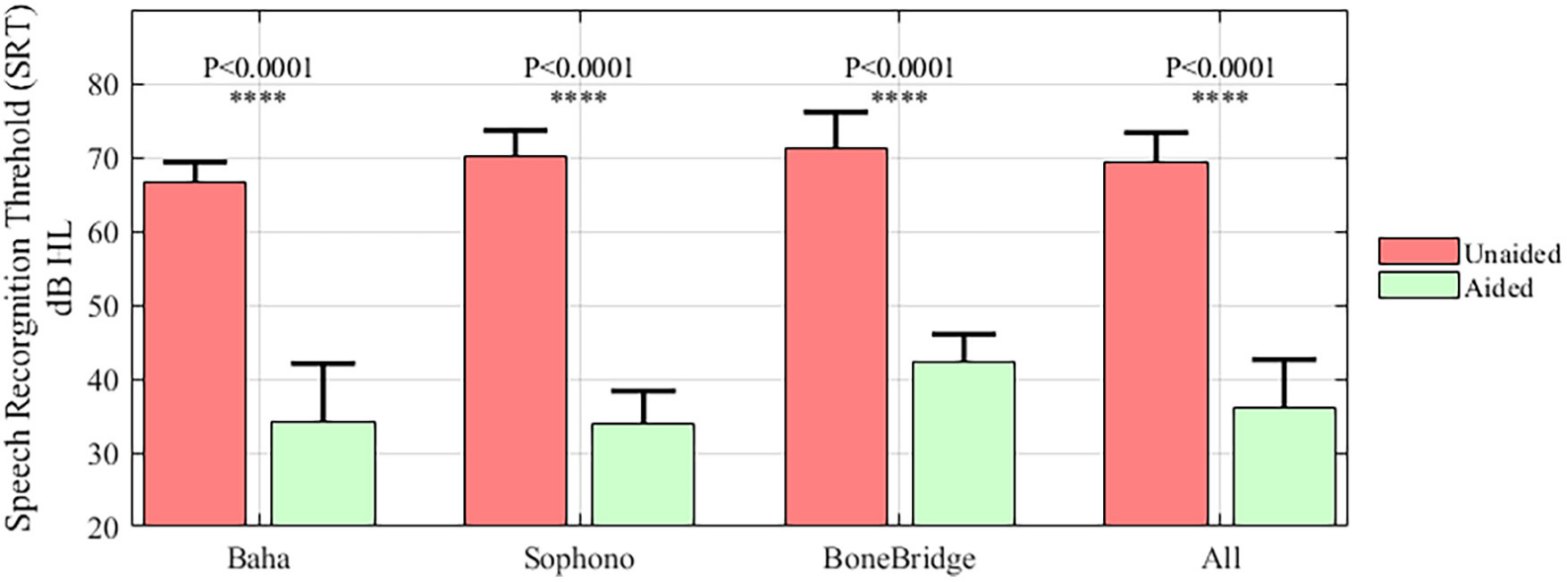
The results of the speech recognition in quiet test for the subgroups and all patients. The results of the unaided condition are shown in red and the aided condition in green.

Compared with the unaided SRT in quiet (69.42 ± 4.21 dB SPL) in all of the participants, the aided SRT (39.16 ± 6.78 dB SPL) significantly improved (*p* < 0.001). The SRT scores of all the subgroups are illustrated in Figure 4. There were no significant differences among the three subgroups regarding the improvement in SRT (unaided SRT minus aided SRT).

### 3.4 Complications

At the end of the follow-up period, no major complications were reported, nor were skin necrosis or infections of the reconstructed ear found. Among all the patients, complications did not greatly impact device fit or wearing.

In the Bonebridge subgroup, the sigmoid sinus of a 9-year-old patient was exposed during the implantation. One patient was found to have a hematoma 5 days after surgery, which was treated by local compression and there were no subsequent adverse events. Another two cases suffered from transient epidermal breakage and soon recovered after reducing the duration of device wearing.

In the Sophono subgroup, complications were reported in two patients. One patient felt pain and swelling in the contralateral ear caused by pressure after the long surgery. The other patient experienced stress-related skin erythema, which was relieved without intervention.

In the Baha Attract group, pressure-related complications, including pain, pitting of the skin, and slight hair loss, were found in 10/13 cases. Those caused by local pressure at the implant site were treated by shortening the duration of using the hearing aid.

## 4 Discussion

### 4.1 The acoustic outcomes

This study presents the acoustic outcomes and safety of unilateral BCD implantation integrated with ear reconstruction surgery. Although restoring bilateral hearing is crucial for achieving functional hearing, the participants in the present study received unilateral BCDs, and bilateral hearing is out of the scope of our study. Indeed, some scholars argue that bilateral BCD implantation may hamper bilateral cues by causing crosstalk (10, 11). Compared with previous clinical studies, the hearing performance of the devices in this study was in accordance with the reported data (12, 13). Even though these implants had similar hearing performance, our data still demonstrated the characteristic hearing outcomes of different implants. In the Baha Attract and Sophono subgroups, which are characterized as transcutaneous BCDs (14), hearing gain at 3 kHz was significantly lower than at the other frequencies (*p* < 0.05). The downsloping aided hearing threshold curve for these two subgroups shows that there was energy attenuation by soft tissue in high frequencies, consistent with previous reports (15, 16). In the Bonebridge group, the representative active BCD, the hearing gain at any two frequencies had no significant differences. However, regarding better performance at high frequencies in free-field pure tone audiometry, the improvement in SRT in the Bonebridge group was not significantly better than the other two groups (*p* > 0.05). Considering that the large volume of the Bonebridge implant makes it unsuitable for patients with microtia with extremely small mastoids, transcutaneous BCD with smaller implants can serve as an alternative. In this case, a preoperative simulation to make an optimal choice of BCD is very important. For patients with microtia presenting with severe mastoid underdevelopment and limited mastoid space, the implantation of a relatively large bone-anchored hearing aid (BAHA) device is challenging. In the case of patients with microtia, a preoperative simulation is essential to confirm the adequacy of the implantation site and mastoid space. In addition, a preoperative trial fitting of bone conduction hearing aids and a comparative assessment of the hearing outcomes with different hearing aids will contribute to a more informed selection of a bone conduction hearing aid for implantation.

### 4.2 Complications

Another factor that affects the decision is the possible complications of the combined surgery. In the present cohort, the implantation procedure did not affect the final result of the plastic surgery. Almost all the complications were device-related. As previously reported, soft tissue intolerance induced by pressure at the implant site was the most common and found mostly in the transcutaneous BCD cases (17). Clinicians should be aware of this when implanting a transcutaneous BCD in a pediatric patient because children do not have the same ability to take care of their scalp at the implant site as adults do. Once skin necrosis, infection, or other major complications occur, the result may be disastrous, resulting in reverse surgery, implant loss, or a second implant (14, 18). Patients and their guardians should be carefully informed about the long-term skin complications related to transcutaneous BCDs, such as Baha Attract, before implantation.

### 4.3 Surgery

In our cohort, there were no complications related to the integrated surgery. Some surgical techniques and postoperative care were vital factors in bringing ideal long-term audiological and esthetic outcomes. The implant must be away from the margin of the incision. Surgeons should keep the proper thickness of the skin above the implants and ensure adequate hemostasis during the operation. A postoperative dressing for the elevated auricle framework and the implant is of paramount importance. At the end of the surgery and during routine dressing changing, a compression dressing should be applied above the implants while keeping very slight pressure around the framework; otherwise, the auricle flap might not survive. The first fitting usually occurs 4 weeks after surgery, and no hematoma or swelling of the skin was found above the implants.

### 4.4 Limitations

The patient sample for each implant in our study was small, and the duration of the follow-ups and age varied considerably between the patients. This may have affected the performance of the pediatric patients during the hearing test and resulted in a conclusion that is not in accordance with other studies. In some studies, Bonebridge has been proven to have better hearing gain than Baha Attract (19). Although other possible solutions, such as the Baha Connect System, Osia, or a non-invasive bone conduction hearing aid, were not examined in this study, they are also suitable for patients with microtia and patients should be informed of them during medical consultation.

## 5 Conclusion

Different BCDs can provide ideal hearing performance for patients with bilateral microtia. Integrating BCD implantation with auricle reconstruction is safe and effective. Aside from the disadvantages of energy attenuation at high frequencies and soft tissue complications, Baha Attract and Sophono are viable alternative choices for those with a small mastoid space.

## Data Availability

The raw data supporting the conclusions of this article will be made available by the authors, without undue reservation.
